# POLICY SERIES: BUILDING TRUST, ADDRESSING BURNOUT AND EXPANDING THE DIRECT CARE WORKFORCE; THE 2022 REPORT TO DHSS AND CONGRESS

**DOI:** 10.1093/geroni/igad104.0692

**Published:** 2023-12-21

**Authors:** Naushira Pandya, Brian Lindberg

## Abstract

This symposium will review the key points and recommendations of the 2022 report to the Secretary of DHSS and Congress, prepared by the federally appointed Advisory Committee on Interdisciplinary and Community-Based Linkages in a blended didactic and interactive format. Clinician burnout following the Covid-19 pandemic is examined and root causes identified. Erosion of the patient trust in the public healthcare system and the consequences thereof are discussed, enhanced role of Community Health workers as the future primary care workforce is explored to prepare the system to prepare for future challenges.

